# Efficacy of an inactivated Zika vaccine against virus infection during pregnancy in mice and marmosets

**DOI:** 10.1038/s41541-021-00426-0

**Published:** 2022-01-27

**Authors:** In-Jeong Kim, Paula A. Lanthier, Madeline J. Clark, Rafael A. De La Barrera, Michael P. Tighe, Frank M. Szaba, Kelsey L. Travis, Timothy C. Low-Beer, Tres S. Cookenham, Kathleen G. Lanzer, Derek T. Bernacki, Lawrence L. Johnson, Amanda A. Schneck, Corinna N. Ross, Suzette D. Tardif, Donna Layne-Colon, Stephanie D. Mdaki, Edward J. Dick, Colin Chuba, Olga Gonzalez, Kathleen M. Brasky, John Dutton, Julienne N. Rutherford, Lark L. Coffey, Anil Singapuri, Claudia Sanchez San Martin, Charles Y. Chiu, Stephen J. Thomas, Kayvon Modjarrad, Jean L. Patterson, Marcia A. Blackman

**Affiliations:** 1 Trudeau Institute, Inc., Saranac Lake, NY 12983 USA; 2 Pilot Bioproduction Facility, Center for Enabling Capabilities, Walter Reed Army Institute of Research, Silver Spring, MD 20910 USA; 3 Southwest National Primate Center, Texas Biomedical Research Institute, San Antonio, TX 78227 USA; 4 Department of Human Development Nursing Science, College of Nursing, University of Illinois Chicago, Chicago, IL 60612 USA; 5 Department of Pathology, Microbiology and Immunology, School of Veterinary Medicine, University of California, Davis, CA 95616 USA; 6 Department of Laboratory Medicine, School of Medicine, University of California at San Francisco, San Francisco, CA 94158 USA; 7 Division of Infectious Diseases, Institute for Global Health and Translational Sciences, State University of New York, Upstate Medical University, Syracuse, NY 13210 USA; 8 Emerging Infectious Diseases Branch, Walter Reed Army Institute of Research, Silver Spring, MD 20910 USA; 9 Present Address: Division of Infectious Diseases and Vaccinology, School of Public Health, University of California at Berkeley, Berkeley, CA 94720 USA

**Keywords:** Viral infection, Viral infection

## Abstract

Zika virus (ZIKV) is a mosquito-borne arbovirus that can cause severe congenital birth defects. The utmost goal of ZIKV vaccines is to prevent both maternal-fetal infection and congenital Zika syndrome. A Zika purified inactivated virus (ZPIV) was previously shown to be protective in non-pregnant mice and rhesus macaques. In this study, we further examined the efficacy of ZPIV against ZIKV infection during pregnancy in immunocompetent C57BL6 mice and common marmoset monkeys (*Callithrix jacchus*). We showed that, in C57BL/6 mice, ZPIV significantly reduced ZIKV-induced fetal malformations. Protection of fetuses was positively correlated with virus-neutralizing antibody levels. In marmosets, the vaccine prevented vertical transmission of ZIKV and elicited neutralizing antibodies that remained above a previously determined threshold of protection for up to 18 months. These proof-of-concept studies demonstrate ZPIV’s protective efficacy is both potent and durable and has the potential to prevent the harmful consequence of ZIKV infection during pregnancy.

## Introduction

ZIKV is a teratogenic pathogen causing severe fetal developmental defects^1,2^. The 2015–2016 outbreak of ZIKV infection in the Western Hemisphere, particularly among pregnant women, was associated with a surge in miscarriages and severe cases of congenital abnormalities, including intrauterine growth restriction (IUGR), microcephaly, and other neuro-developmental disorders, referred to as congenital Zika syndrome (CZS)^2–6^. Consequently, in February 2016, the World Health Organization declared the ZIKV epidemic to be a public health emergency of international concern^7,8^. As such, the development of a ZIKV vaccine became an urgent public health priority. Despite the greatest need for the vaccine in pregnant women, this population is typically reserved for the final stages of clinical evaluation, given concerns for potential unforeseen adverse effects on the mother and developing fetus^9–13^. Inactivated virus vaccine platforms, however, have a long track record of safety in both pregnant women and fetuses^14^. For example, vaccination with inactivated influenza virus vaccines^15^ is recommended for pregnant women, as the benefits have been shown to outweigh potential risks.

The Zika purified inactivated virus (ZPIV) vaccine is a whole formalin-inactivated ZIKV derived from the Puerto Rico (ZK-PR) strain, PRVABC59, developed by the Walter Reed Army Institute of Research (WRAIR). The vaccine is co-formulated with aluminum hydroxide adjuvant. Preclinical studies in mice and rhesus macaques have shown that ZPIV-induced virus-neutralizing antibodies protected against viremia after ZIKV challenge^16,17^. Protective immunity persisted for at least 1 year after vaccination in non-human primates (NHPs)^18,19^. In addition, ZPIV has been shown to be safe and immunogenic in humans in phase 1 clinical trials^20,21^ and induced cross-protective B cell responses in human against Zika and dengue viruses^22^. However, the protective efficacy of ZPIV has not been established in the priority population, women of child-bearing age and pregnant women. In this study, we examined proof of concept that a ZIKV vaccine candidate could protect the unborn fetus in two immunocompetent pregnant animal models: C57BL/6 mice and common marmoset monkeys (*Callithrix jacchus*).

Previously, it was shown that ZIKV infection during pregnancy in wild-type C57BL/6 mice resulted in placental insufficiency and fetal demise^23^. Thus, pregnant C57BL/6 mice may be a high-fidelity model for non-productive ZIKV infection-induced fetal malformations; a model that may be suitable for an initial evaluation of vaccine efficacy. NHPs, however, are more physiologically relevant models for exploring vaccine efficacy in pregnant women because of the similarities in their placental structure and gestational period^24–28^. Common marmosets are susceptible to infection with flaviviruses, including dengue virus^29–31^ and ZIKV^32–34^. It previously has been shown that ZIKV infection during pregnancy in marmosets caused trans-placental virus transmission and led to spontaneous abortion within 16 days of infection^34^. Therefore, marmosets provide clinically relevant models to study the protective efficacy of vaccines against *in utero* ZIKV infection and trans-placental ZIKV transmission. An additional feature of marmoset biology that enhances their utility as pregnancy models for evaluating prophylactic interventions during pregnancy is their high frequency of multiple births including twins, triplets, and quadruplets^35^. In this report, we have exploited the relative strengths of these immunocompetent mouse and marmoset models, to test the protective efficacy of ZPIV following ZIKV challenge during pregnancy.

## Results

### Protection by ZPIV against fetal abnormalities caused by ZIKV infection during pregnancy

**Fig. 1:**
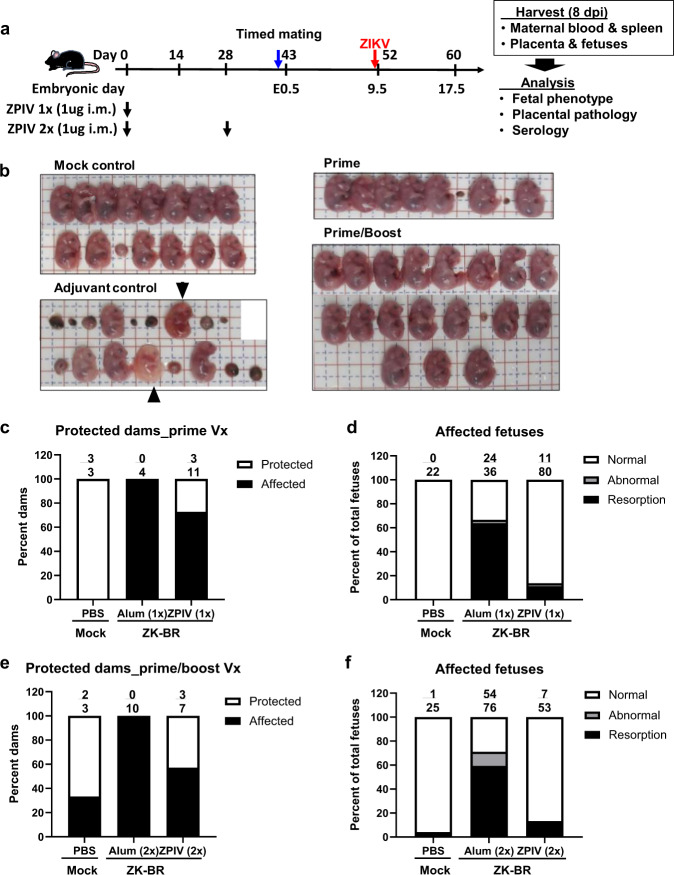
Protective effect of ZPIV after prime or prime/boost vaccination. **a** The experimental scheme. Six-week-old female wild-type C57BL/6 mice were intramuscularly (i.m.) injected with alum adjuvant alone, or 1 ug alum-adjuvanted ZPIV with either a one (at 0 week) or two (at 0 and 4 weeks) dose regimen. Two weeks after the last vaccination, females were mated and challenged at embryonic day 9.5 (E9.5) with 6 × 10^5^ PFU ZK-BR and examined at 8 dpi (E17.5). **b** Representative images of fetal phenotypes per group. The horizontal and vertical lines in the grid mark 5 mm intervals. Note macerated fetuses with abnormal vascular development (arrowheads) and extensive fetal demise after ZIKV challenge of mice that received adjuvant alone. The percentage of dams bearing fetuses with normal phenotype after prime (**c**) or prime/boost (**e**) vaccination. The numbers above the individual bar indicate the number of dams without any fetal abnormality over the total number of dams examined per group. The percentage of affected fetuses of total fetuses per group after prime (**d**) or prime/boost (**f**) vaccination. The numbers above individual bars indicate the number of affected fetuses over the total number of fetuses per group. The percentage of fetal abnormality was analyzed using Fisher’s exact test. The difference between the alum only and the ZPIV vaccinated group was significant, *P* < 0.0001 but not between the mock-infected and the prime/boost vaccinated groups (*P* > 0.05).

We first tested ZPIV’s efficacy in preventing gross morphological defects and fetal abnormalities in C57BL/6 mice following heterologous infection with the Brazilian strain of ZIKV, Brazil SPH2015 (ZK-BR), during pregnancy (Fig. 1a, b). Efficacy was evaluated in terms of reduction of the number of dams (pregnant female mice) bearing fetuses with morphological defects (Fig. 1c) and the percentage of affected fetuses (Fig. 1d). The data show that 100% of ZIKV infected unvaccinated dams bore affected fetuses, whereas a single vaccine dose, 1 ug ZPIV, protected three out of 11 dams (27%) from bearing affected fetuses (Fig. 1c). A single-dose ZPIV was even more pronounced in its efficacy when measuring malformed fetuses-significantly reducing the proportion of affected fetuses from 67% (24 out of 36) in the unvaccinated group to 13.8% (11 out of 80) in the vaccinated group (Fig. 1d, *P* < 0.0001), resulting in a relative efficacy of ZPIV in preventing fetal malformations of 79.4% (Supplementary Table 1).

An important question was whether a prime-boost regimen would confer additional protection over the single-dose regimen. Interestingly, a second vaccine dose at 28 days after prime reduced the proportion of affected fetuses from 71.1% (54 out of 76) in the unvaccinated group to 13.2% (7 out of 53) after prime-boost vaccination, of which the relative efficacy was slightly improved, compared to the single-dose regimen, to 81.4% (Supplementary Table 1 and Fig. 1f). These results showed that both single-dose and two-dose ZPIV vaccination provided approximately 80% efficacy in the prevention of fetal malformations induced by ZIKV infection. Previously, humans were vaccinated with two doses of ZPIV^20,21^. In order to maintain consistency with the human clinical trials, we used the prime-boost vaccination regimen to examine the efficacy of ZPIV in pregnant animal models for the rest of the study.

### Cross-strain protection by ZPIV in mice

**Fig. 2:**
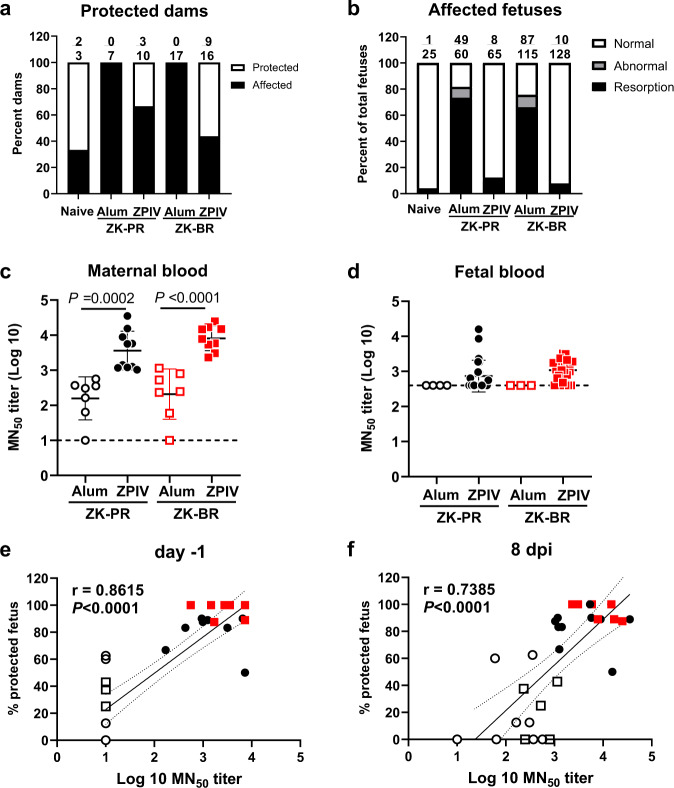
Protection by ZPIV against homologous and heterologous ZIKV challenge during pregnancy. Adult female C57BL/6 mice were i.m. immunized with two doses of either alum only (clear symbol) or ZPIV (solid symbol) as described in Fig. 1. After establishing pregnancy, the mice were challenged with either the ZK-PR strain or the ZK-BR strain at E9.5 and examined at 8 dpi (E17.5). **a** The percent protected dams carrying all fetuses with normal phenotype over the total number of dams. **b** The percent affected fetuses, based on gross fetal phenotype, of total fetuses. Gray regions indicate the fraction of phenotypically abnormal fetuses excluding those with complete fetal demise (solid black). The frequency of fetal demise in vaccinated mice was significantly reduced (*P* < 0.0001) using Fisher’s Exact test, compared with that of the group with alum alone. At 8 dpi, virus-neutralizing antibodies were determined as MN_50_ titers (mean ± S.D.) in maternal serum samples (**c**) and fetal serum samples (**d**). Symbols represent individual mice challenged with ZK-PR (circle) or ZK-BR (square). The number of fetuses examined are open circles, *n* = 4; closed circles, *n* = 24; open squares, *n* = 3, closed squares, *n* = 27. The Kruskal-Wallis test was used for statistical analysis. The dotted line indicates the lowest serum dilution examined, which was 1:10 (**c**) and 1:400 (**d**). The percentage of fetal protection at 8 dpi was plotted against log10 MN_50_ titers in maternal serum samples from alum only (clear circle, *n* = 13) and vaccinated (solid circle, *n* = 19) mice with combined groups challenged with ZK-PR or ZK-BR, as shown in Supplementary Table 3, at day −1 (**e**) and 8 dpi (**f**). The correlation was determined using the Spearman *r*-test. Spearman’s correlation coefficient, *r* = 0.8615 with 95% confidence interval (C.I.; dotted line), 0.733–0.931 in **e** and *r* = 0.7385 with the 95% C.I., 0.525–0.865 in **f**. The values were statistically significant, *P* < 0.0001.

ZPIV is based on the Puerto Rico strain of ZIKV, PRVABC59. We examined the protection of mice against infection with ZK-PR and ZK-BR strains. The purpose of examining protection against both virus strains was two-fold: the first was to make a seamless transition from mouse to marmoset studies because, in the previous study, infection with ZK-BR induced abortion in marmosets^34^, and the second was to determine whether ZPIV can elicit cross-protection against these two viral strains. Challenge with ZK-PR or ZK-BR strains in two-dose ZPIV vaccinated mice yielded the relative efficacy of 84.9% and 89.7%, respectively (Supplementary Table 2), demonstrating that ZPIV vaccination provided comparable cross-strain protection against two ZIKV strains (Fig. 2).

### Correlation of neutralizing antibody titers and protection of the fetus in mice

At 2 weeks after primary vaccination, the geometric mean of neutralizing antibody levels (log10 MN_50_ titer) was 1.46 (with 95% confident intervals (C.I.) 1.26–1.69). Subsequently, the neutralizing antibody titer was 100- fold increased (geometric mean of 3.52 with 95% C.I. 3.34-3.72) at 2 weeks after the boost (6 weeks after prime, Supplementary Table 3), which was significantly different from the levels after primary vaccination (*P* < 0.0001). In the vaccinated groups, the maternal virus-neutralizing antibody titers at −1 (7 weeks post-prime) and 8 days after infection (8 dpi, 8 weeks post-prime) were comparable (Supplementary Table 3 and Fig. 2c). ZIKV neutralizing antibodies were also detected in the fetal blood collected at embryonic day 17.5, 8 dpi. Due to the limited fetal blood volumes, the samples were tested at a high dilution, 1:400, the limit of detection with the fetal serum samples. Fetal neutralizing antibody levels in the unvaccinated groups were all at the limit of detection, whereas nine out of 23 fetuses (39%) and 19 out of 24 fetuses (79%) from the vaccinated groups challenged with either ZK-PR or ZK-BR, were above the limit of detection (Fig. 2d). The proportion of phenotypically normal fetuses per litter determined at 8 dpi positively correlated with the neutralizing antibody levels at 1 day before (Fig. 2e) and 8 days after (Fig. 2f) virus challenge. One day before ZIKV infection, the majority of vaccinated dams (17 out of 19) with >2.4 log10 MN_50_ titers protected 80% of the fetuses per dam (e.g., phenotypically normal fetuses, six out of an average litter size of eight). These results support that the vaccine-elicited neutralizing antibodies play a critical role in the protection of fetuses against CZS.

### Protection by ZPIV against ZIKV infection during pregnancy in marmosets

**Fig. 3:**
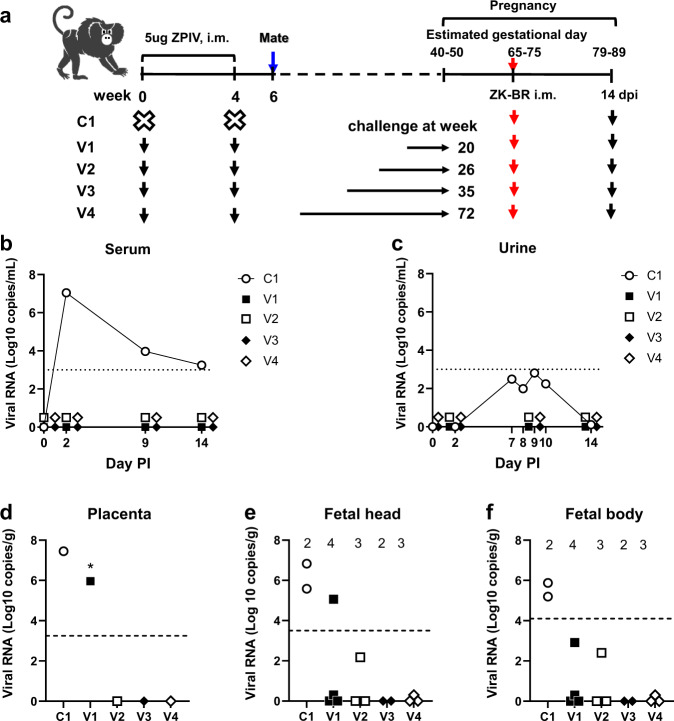
ZPIV reduced viral RNA levels in fetal tissues in marmosets. The marmoset study scheme. Four marmosets, V1–V4, were i.m. injected with two doses of 5 ug alum/ZPIV 4 weeks apart. After co-housing with males, pregnancy and estimated gestational day (EGD) were determined by ultrasound examination. Individual marmosets became pregnant at different times after vaccination. Time-lapse between the last dose of vaccine and the time of virus challenge is indicated as a dotted line in (**a**). Unvaccinated control C1 and vaccinated V1–V4 marmosets were i.m. injected with two doses of 2.5 × 10^5^ PFU ZK-BR 4 days apart during EGD 65–76 corresponding to 20, 24, 36, or 72 weeks after prime vaccination. Serum and urine samples were prepared at 0, 2, 9, and 14 days after infection (dpi) and viral RNA loads were determined in serum (**b**) and urine (**c**) using Real-Time qRT-PCR. All marmosets were sacrificed for examination at 14 dpi (EGD 79–89) and viral RNA levels in the placenta (**d**), fetal head (**e**), and fetal body (**f**) were determined. Three different locations in the placenta were sampled. RNA from each placental sample was prepared independently, and each sample was tested in duplicate. The asterisk indicates that one out of three locations in the placenta was positive for viral RNA (2.7 × 10^7^ copy/g tissue) while the other two sites were negative for viral RNA. The mean of the three locations is presented. The number of fetuses per pregnant marmoset at each time point is indicated above the symbols in (**e**) and (**f**). The horizontal dotted lines indicate the limit of quantification per tissue, as described in Materials and Methods.

We further examined the ability of ZPIV to prevent ZIKV transmission from mother to fetus during pregnancy in the litter-bearing NHP model of common marmosets. Prior to mating, we immunized four female marmosets, V1–V4, with two doses of 5 ug ZPIV 4 weeks apart. An additional female marmoset served as an unvaccinated control, C1 (Fig. 3a). Pregnancy was monitored via ultrasound. All pregnant marmosets were challenged at estimated gestational day (EGD) 65–75 during the second trimester, and maternal and fetal tissues were collected for examination at 14 dpi in the late second trimester. All marmosets in this study received ZPIV at the same time and were challenged with ZIKV at a comparable gestational time during pregnancy. However, because vaccinated marmosets became pregnant at different times, individual V1–V4 marmosets were challenged at 20, 26, 36, or 72 weeks after vaccination, respectively. This presented the opportunity to explore the durability of ZPIV immunogenicity and protective efficacy.

In control marmoset C1, viral RNA in serum and urine samples was detectable during the acute phase and subsided by 14 dpi, whereas viral RNA was not detected in serum or urine at any time point after ZIKV infection examined in the vaccinated marmosets, V1–V4 (Fig. 3b, c).

In control marmoset C1, relatively high viral RNA levels were detectable in maternal spleen, mesenteric lymph nodes, placenta, and fetal tissues (Table 1). However, immunohistochemistry was unable to detect viral proteins in these maternal and fetal tissues in C1. The primary goal of this study was to determine the ability of ZPIV to prevent vertical transmission. At 14 dpi, 11 of 12 fetuses from V1–V4, regardless of different times after vaccination, were protected, as viral RNA was either not detected or was below the limit of quantitation (BLQ, Ct value > 35) in the placenta, fetal heads and fetal bodies (Table 1 and Fig. 3d–f). One of the quadruplets from the vaccinated marmoset V1 challenged at 20 weeks after vaccination showed a viral RNA load at 1.2 × 10^5^ copies per gram (the average Ct value of 32.1) in the head. Viral RNA loads in the other fetuses were either BLQ or not detectable. However, a substantial level of viral RNA (2.52 × 10^7^ copies per gram tissue) was detected in one of three randomly sampled sites from the placenta of V1, but not in V2–V4. We tested the placental homogenate to examine whether viral RNA was from infectious particles or fragments of viral genome from destroyed virus. By the standard focus forming assay, infectious virus particles were not detected in placental homogenates from C1 and V1. To increase the sensitivity of detecting infectious virus particles, placental homogenates were co-cultured with human monocyte U937 cells. If infectious virus particles have formed immune complexes with anti-ZIKV antibodies in the placenta, the virus would presumably gain access to host cells via Fcγ receptors. Virus-infected U937 cells were detected after incubation with placental homogenate from the unvaccinated ZIKV infected marmoset C1 but not in the cells incubated with the homogenate from naïve or vaccinated marmosets (Supplementary Fig. 1), confirming that the PCR products detected in the placenta of the marmoset V1 were not derived from infectious virus particles. Although all marmosets were infected with ZIKV at a comparable gestational day (65–75), because vaccinated marmosets became pregnant at different times, individual marmosets were challenged at 20, 26, 36, or 72 weeks after vaccination. Therefore, the results indicated that ZPIV-induced immunity was protective and durable, lasting up to 18 months after vaccination.

**Table 1:** ZIKV RNA loads in maternal and fetal tissues in marmosets^a^.

ID	Treatment	Challenge^b^	Fetus^c^	F. Hd^d^	F. Bd^e^	Placenta	M. Spl^f^	M. mLN^g^
C1	None	0	2/2	4–67	2–7.5	284	15.5	2120
V1	ZPIV 2x	20	1/4	1.2	BLQ^h^	+/− ^i^	0.0^j^	0.0^j^
V2	ZPIV 2x	26	1/3	BLQ^h^	BLQ^h^	BLQ^h^	0.0^j^	0.0^j^
V3	ZPIV 2x	36	0/2	0.0^j^	0.0^j^	0.0^j^	0.0^j^	0.0^j^
V4	ZPIV 2x	72	0/3	0.0^j^	0.0^j^	0.0^j^	0.0^j^	0.0^j^

^a^ZIKV RNA load is presented as indicated number multiplied by 10^5^ copy per gram tissue.

^b^All marmosets were i.m. challenged with ZIKV-BR at indicated weeks after prime dose vaccination.

^c^Number of ZIKV RNA-positive fetuses per total number of fetuses.

^d^F. Hd, fetal head.

^e^F. Bd, fetal body.

^f^M. Spl, maternal spleen.

^g^M. mLN, maternal mesenteric lymph nodes.

^h^Samples contained detectable signals for viral RNA; however, the level was below the limit of quantitation (BLQ, Ct value > 35) as described in Materials and Methods.

^i^Placental tissues were sampled at three different locations, RNA was isolated from each piece separately, tested for RT-PCR in duplicate of each origin of tissue. One site was positive (2.52 × 10^7^ copy g^−1^ tissue) for ZIKV RNA whereas the other two sites were negative. No infectious virus particles were detectable after co-culture of placental homogenates with Vero cells and U937 cells (Supplementary Fig. S1).

^j^Viral RNA was not detectable and considered below the limit of detection (Ct value > 37). The tissue tpe-specific limit of detection (log10 copy number g^−1^) are 3.8 in the fetal head, 4.2 in the fetal body, 3.4 in the placenta, 4.11 in the spleen, and 3.08 in the LN.

### ZPIV elicited neutralizing antibodies in marmosets

Neutralizing antibodies in the vaccinated marmosets ranged between 2.2 and 2.9 log10 MN_50_ titers before ZIKV infection and increased approximately 10-fold at 9 dpi (Table 2). Comparison of neutralizing antibody titers prior to ZIKV infection among vaccinated marmosets showed that log10 MN_50_ titers >2.5 prevented vertical transmission, indicated by the absence of detectable viral RNA in the fetal tissue at 14 dpi. The neutralizing antibody titer of marmoset V1 with partial protection was relatively lower (2.24 log10 MN_50_) than those in V2–V4. These data show that high neutralizing antibody titers elicited by ZPIV contribute to the protection of mothers and fetuses, and are consistent with a prior study showing that the protection of rhesus macaques (six out of eight) against ZIKV challenge correlated with neutralizing antibody titers >2.0 log10 MN_50_ titers^18^.

**Table 2:** Virus-neutralizing antibody (MN_50_) titers in marmosets before and after ZIKV challenge^a^.

ID	Treatment	0 week^b^	2 weeks^b^	6 weeks^b^	Challenge^c^	-7 to -5^d^	9 dpi^d^	14 dpi^d^
C1	None	N.A^e^	N.A^e^	N.A^e^	0	0.7	1.56	3.26
V1	ZPIV 2x	0.7	1.81	3.68	20	2.24	4.79	>3.86^f^
V2	ZPIV 2x	0.7	2.36	N.T^g^	26	2.95	3.80	>3.86^f^
V3	ZPIV 2x	0.7	1.64	3.61	36	2.60	3.70	>3.86^f^
V4	ZPIV 2x	0.7	2.50	3.70	72	2.77	4.33	4.90
GMT^h^	–	0.7	2.05	3.72	–	2.62	4.13	4.1
95% C.I^i^	–	(0.7)	(1.5–2.8)	(3.4–4.1)	–	(2.2–3.2)	(3.4–5.0)	(3.4–4.9)

^a^Log-transformed reciprocal serum dilution achieving 50% neutralization of virus.

^b^Weeks after prime dose vaccination.

^c^All marmosets were i.m. challenged with ZIKV-BR at indicated weeks after prime dose vaccination.

^d^Days after infection with ZIKV-BR.

^e^N.A., Not applicable.

^f^The last serum dilution achieved >50% of neutralization.

^g^N.T., Not tested.

^h^GMT, geometric mean.

^i^C.I. confidence interval.

## Discussion

The ability of ZIKV to cross the placental and blood-brain barriers to cause severe neurological disorders in developing fetuses makes the development of vaccines to prevent CZS an urgent global health priority. Ideally, a ZIKV vaccine would completely prevent vertical transmission of ZIKV across the placenta and the resulting clinical manifestations of CZS. The most recent ZIKV outbreak in the Americas did not afford the opportunity to establish protective efficacy in humans or safety and efficacy in pregnant women. Therefore, we attempted to measure the efficacy of vaccines using experimental pregnancy models. We demonstrated the efficacy of the ZPIV vaccine candidate in preventing fetal abnormality and vertical transmission of ZIKV during pregnancy in both immunocompetent mice and marmosets.

We previously reported fetal demise after ZIKV infection during pregnancy in immunocompetent C57BL/6 mice that were not associated with direct infection of the fetus, but with placental insufficiency^23^. Despite this, the fetal demise induced by ZIKV infection was dose-dependent. Therefore, this pregnant mouse model offered an initial, experimentally amenable, testing platform to evaluate the ability of vaccines to prevent ZIKV-associated fetal demise and malformations. We also previously reported that ZIKV infection during marmoset pregnancy caused spontaneous fetal abortion associated with vertical transmission of virus^32^. This provided a second, more physiologically relevant, NHP pregnancy model to test vaccine efficacy.

In the current study, both prime and prime-boost vaccination of ZPIV provided 80% efficacy in protection against fetal malformations after ZIKV infection during pregnancy in C57BL/6 mice. Previously, an intramuscular (i.m.) injection of a single dose of ZPIV was shown to provide complete prevention of viral infection in non-pregnant Balb/C mice^17^. Different study conditions between the current and previous studies may stem from several factors: (1) differing susceptibility to infection and pathogenicity of different mouse strains, (2) the higher viral dose (5 × 10^5^ PFU) used for challenge in this study compared to the published studies (10^2^ PFU)^17^, (3) different quantities of neutralizing antibodies might be required for protecting pregnant versus non-pregnant mice, and (4) different readouts for protection in the different models—viral titer versus fetal abnormality. Despite these differences, taken together, the two studies support the protective efficacy of ZPIV- both for preventing infection in non-pregnant mice and also for preventing fetal sequelae following infection during pregnancy.

Importantly, neutralizing antibody titers correlated with protection of dams and fetuses against ZIKV infection. Previously, ZPIV studies showed that passive transfer of the immune sera with neutralizing activity >2 log10 MN_50_ titers from rhesus macaques or humans to naïve Balb/C mice conferred protection against ZIKV challenge^16,18^. In the current study, we showed that >80% protection of the fetuses per litter in pregnant C57BL/6 mice was achieved by log10 MN_50_ titers >2.4. These results agreed with the results from Shan et al.^36^ that higher titers of neutralizing antibodies were required for protection against intrauterine infection in pregnant mice versus protective titers in non-pregnant mice. Together, these study results strongly support that neutralizing antibody titers correlated with the protection of fetuses against ZIKV-induced fetal demise. In addition, ZPIV was shown to produce broadly cross-neutralizing antibodies against four different ZIKV strains derived from the Asian and African lineages in Balb/C mice^18^. In agreement, our current study results confirmed that the ability of ZPIV to cross-protect against the ZK-PR and the ZK-BR infection during pregnancy.

Previous studies showed that maternal infections in marmosets during the second trimester rendered fetal cortical brain infection including optical nerve cells and disorganized migrating neurons in the cortical plate recapitulating key features of neuroinvasive infections^32,34^ that were not afforded by other NHP models. Importantly, marmosets have two specific advantages over rhesus macaques as an experimental pregnancy model^37^; first, marmosets breed year-round whereas rhesus macaques breed seasonally, and, second, marmosets give multiple births (litter size 2–4) per pregnancy^35^, which increases sample size and hence increases the statistical power of data analysis. Other experimental advantages of marmoset over rhesus macaques include the fact that marmosets are not susceptible to human hepatitis B virus; therefore, there are fewer animal care concerns, and, the small-size (300–500 g) of marmosets increases the capacity of animal holding and maintenance per unit when compared to rhesus macaques, reducing the cost of the animal care and maintenance. The marmoset studies reported here showed that vaccination induced high neutralizing antibody titers and prevented vertical transfer of virus in 11 out of 12 fetuses. The neutralizing antibody titer before ZIKV infection in the marmoset with incomplete protection was relatively lower (2.24 log MN_50_ titer) than the protected marmosets (>2.5 log MN_50_ titers). Our studies also address the duration of vaccine protection. Because the pregnancies of the vaccinated marmosets were not synchronized in the current study, viral challenge occurred at different time points after vaccination, allowing us to assess the longevity of the protection from 20 to 72 weeks after vaccination. This result showed that ZPIV provided protection for up to 72 weeks after vaccination in marmosets, suggesting that the vaccine establishes a durable protective memory B cell response. Regardless of the time-lapse between vaccination and ZIKV challenge, neutralizing antibodies >2.5 log MN_50_ titer prior to challenge confer prevention of vertical transmission, underscoring the role of neutralizing antibodies in protection against virus infection.

In the current study, we only measured the neutralizing activity of antibodies. Non-neutralizing antibodies may also contribute to anti-viral immunity, through antibody-dependent cell-mediated cytotoxicity and/or antibody-dependent cellular phagocytosis. In addition, the affinity and avidity of antibodies, which were not investigated in this study, may affect the protective effects. Finally, both ZPIV and an optimized version of ZPIV, ZPIV-SP, have been shown to elicit not only a ZIKV-specific antibody response but also cell-mediated immune responses in NHPs^18,19^. T cells producing IFNγ and/or IL-5 upon stimulation with the envelope protein of ZIKV may contribute to the protection, either by providing T helper signals to memory B cell generation or by stimulating cytotoxic T cell activity.

ZPIV also has been shown to be safe in healthy non-pregnant humans^20,21^. Because of its expected safety profile, inactivated virus vaccines, including ZPIV adjuvanted with aluminum hydroxide, would be a potentially favored platform for vaccinating pregnant women. This aspect was not examined in the current study. Further preclinical studies are necessary to investigate the efficacy of ZPIV vaccination during pregnancy. An additional complexity of ZIKV vaccination not examined in this study is the potential effect on vaccination efficacy of co-circulating flaviviruses. How the interplay between ZIKV immunity and other flaviviruses might impact vaccine efficacy remains unclear and would need to be explored in the context of large-scale clinical end-point trials^38,39^. Here, we have used preclinical pregnancy models to show the protective efficacy of ZPIV against ZIKV infection during pregnancy by its ability to inhibit viral infection and prevent ZIKV-associated congenital defects. Our studies demonstrate proof of concept in animal pregnancy models that a vaccine has the potential to protect the unborn fetus from ZIKV infection and that at least one immunological mechanism of protection is vaccine-induced neutralizing antibody.

## Methods

### Ethics statement

All animal studies were conducted in accordance with the Guide for Care and Use of Laboratory Animals of the National Institutes of Health. Prior to initiation, all mouse studies were conducted at Trudeau Institute following the approved Institutional Animal Care and Use Committee (IACUC) protocol. Marmosets were housed at the Southwest National Primate Research Center (SNPRC), at Texas Biomedical Research Institute. All marmoset studies were reviewed and approved by the local IACUC, and Biohazard Committee prior to initiation. All mouse and marmoset studies were performed following the protocols approved by IACUC at the performing sites and by the Animal Care and Use Review Office (ACURO) in compliance with the Animal Welfare Regulations (AWRs), the *Guide for the Care and Use of Laboratory Animals*, required for the Federal and the Department of Defense regulations.

### Mouse studies

Six-week-old wild-type C57BL/6 J mice were purchased from the Jackson Laboratory (Bar Harbor, ME) and housed at Trudeau Institute. Mice were intramuscularly injected with either one or two doses of alum alone or 1 ug alum-adjuvanted ZPIV 4 weeks apart prior to mating (*N* = 8 per group). Then, at the 6 week time point (2 weeks after boost), female mice were primed for breeding by introducing bedding from male cages 2–3 days prior to mating and then co-housed with male in a 1:1 ratio per cage. Females were checked daily for the detection of a copulatory plug (E0.5) as described previously^23^. At embryonic day 9.5 (E9.5), C57BL/6 mice were anesthetized with isoflurane and injected with 100 uL of 6 × 10^5^ PFU ZK-BR or 5.67 × 10^5^ PFU ZK-PR via the retro-orbital sinus vein and euthanized 8 days after infection (E17.5) by CO_2_ inhalation with the flow rate at 4 L per minute for at least 60 s until the mouse is no longer breathing following Standard Operation Protocol as described previously^23^. Maternal blood was collected via cardiac puncture. Using aseptic technique, maternal spleen and uterus were removed, and fetuses and placentas were separated. Randomly selected 2–3 fetuses per dam were placed in tissue cassettes and immersed in 10% neutral buffered formalin (NBF) for histology. The rest of the fetuses were decapitated and fetal blood samples were collected in sterile Eppendorf tubes. Fetal heads and bodies were snap frozen in liquid nitrogen and kept at −80 °C until further processed for RNA isolation.

### Marmoset studies

All marmoset studies were conducted at the Texas Biomedical Research Institute. General husbandry, diet and enrichment for the colony at SNPRC has been previously described^40,41^. Six adult nulliparous females between 2–2.5 years old with an average weight of 485 g (±40 g) were enrolled in the study. The male partners were between 3–4 years old. The females were singularly housed during the 8-week vaccination period within visual, auditory, and olfactory range of the other adult females and males in the room.

Four female marmosets (V1–V4) received two, 5 ug intramuscular injections of ZPIV in aluminum hydroxide 4 weeks apart and co-housed with a male at 4 weeks after the last dose. The control female (C1) was left untreated and challenged with ZIKV at comparable gestational days. One additional pregnant female also served as a gestational day comparable naïve control for comparison of the development of the placenta. The gestational age of the embryos was estimated using crown-rump length assessed via ultrasound, which has previously been found to reliably estimate gestational age in marmosets to within ±3 days^42,43^. Following vaccination, each female received an ultrasound without sedation each month until pregnancy was confirmed. Pregnancy was typically detected at ~30 days gestational age. The female received a second ultrasound 21–28 days after pregnancy detection to confirm the gestational age of the pregnancy. Females were intramuscularly inoculated with Zika virus twice during the second trimester (day 65–75 of gestation). Ultrasounds were also performed at day 9 and 14 post inoculation to monitor fetal development.

At 14 dpi, females were sedated with ketamine (15 mg/kg) and euthanized by an i.v. injection of 0.5–1.0 ml Fatal Plus (Vortech Pharmaceuticals) at necropsy to yield placenta and fetuses between the estimated gestational day of 79–89, average of day 86 (late second trimester). The placenta was removed from the uterus using sterile technique. The placental disks were separated and each fetus was removed. The number of fetuses ranged from 2–4 at the time of collection. The head of each fetus was removed and cut in half along the sagittal plane. One half of the fetal head was weighed and snap frozen in liquid nitrogen immediately and the other half was placed in a cassette in 10% NBF. The fetal body was weighed and snap frozen in liquid nitrogen. All frozen tissues were stored at −80 °C until processed. Each disc of the placenta was measured and photographed. One disc was weighed intact and placed in 10% NBF for stereological analysis, the other disc was divided into four equal pieces with two pieces of the disc weighing 0.03 to 0.1 g each being snap frozen, the other pieces were placed into a cassette and stored in 10% NBF for histology.

### Viruses, cells, and titration

The ZIKV-BR SPH2015 strain was obtained from Dr. Lark Coffey (UC-Davis). The strain was passaged two times (P2) after isolation from a patient in Brazil in 2015^44^. Without further propagation, the P2 stock was used to inoculate marmosets at Texas Biomedical Research Institute and a vial was sent to Trudeau Institute. At the Trudeau Institute, P2 was propagated twice more in Vero cells (P4). Then, P4 was used for inoculating mice. Therefore, virus stocks used for marmosets and mice differ by two passages. Sequence analysis was performed initially at UC-Davis. Using the sequence derived at UC-Davis as a reference, deep-sequence analyses of P4 was performed at UCSF (Supplementary Table 4).

The ZIKV Puerto Rico strain PRVABC59 (ZK-PR) was received from BEI Resources, NIAID, NIH. Vero cells (African green monkey kidney epithelial cells) were purchased from American Type Culture Collection (ATCC, CCL-81), and maintained in DMEM supplemented with 10% heat-inactivated FBS, 2 mM L-glutamine, 1 mM penicillin–streptomycin and 0.3% sodium bicarbonate at 37 °C with 5% CO_2_. Viral titers of the stock were determined by plaque assays and focus forming assays on Vero cells as described previously^23,45^. The virus was thawed, diluted to the appropriate plaque forming units with 1x PBS. Marmosets received two intramuscular injections (2.5 × 10^5^ PFU/50 uL per dose) of the ZK-BR strain between 65–75 days of gestation 4 days apart. At embryonic day E9.5, C57BL/6 mice received intravenous injections of 5.67 × 10^5^ PFU ZK-PR or 6 × 10^5^ PFU ZK-BR, that were previously determined to cause approximately 70% fetal growth defects.

### Vaccine (WRAIR)

ZPIV vaccine was developed, prepared, and provided to all sites by WRAIR. ZPIV contains a chromatographic-column-purified, formalin-inactivated Zika virus Puerto Rico strain (PRVABC59) that was initially obtained from the Centers for Disease Control and Prevention (Fort Collins, CO, USA) and cultured and passaged in a qualified Vero cell line. After purification and inactivation, ZPIV was absorbed in a 1:1 ratio with 1 mg/mL alum (Alhydrogel, Brentagg Biosector, Frederikssund, Denmark). Alum-absorbed ZPIV was prepared at the concentration of 10 μg/mL.

### Microneutralization (MN_50_) assay (WRAIR)

Neutralizing antibody titers were determined using a high-throughput ZIKV microneutralizing antibody assay at WRAIR, as previously described^17,18^. Briefly, all serum samples were heat-inactivated at 56 °C for 30 min. All maternal serum samples from mice and marmosets were prepared at 1:10 dilution, the highest concentration, and further diluted using 3-fold dilution series. Eight dilutions were tested for neutralization. For mouse fetal serum samples, due to limited volumes obtained at E17.5 (8 dpi), samples were prepared at 1:400 dilution as the highest concentration then serially diluted. Then, maternal and fetal serum samples were further diluted using 3-fold dilution series, mixed with 100 PFU of ZV-PR (PRVABC59) per well, and incubated at 35–37 °C for 2 h. The mixtures were added to 96-well plates containing confluent Vero cell monolayers in triplicate wells. Following the incubation for 4 days, the plates were washed and fixed in a 1:1 mixture of absolute ethanol and methanol for 1 h at −20 °C. After washing and blocking, the plates were incubated with pan-flavivirus monoclonal antibody clone, 6B6-C1 (a gift from J. T. Roehrig, U.S. Centers for Disease Control and Prevention) conjugated with HRP for 2 h. The plates were then washed and incubated with TMB substrate for 50 min at RT. The enzymatic reaction was stopped by adding1:25 phosphoric acid, and the absorbance was measured optical density (OD) at 450 nm. Fifty percent microneutralization (MN_50_) titers were determined as the reciprocal serum dilution corresponding to the wells reducing OD values by 50% when compared with that of the wells containing 100 PFU of virus alone based on a standardized assay protocol for other licensed flavivirus vaccines.

### Real-time qRT-PCR

Frozen tissue samples were added to RLT buffer (QIAGEN) containing β-mercaptoethanol (β-ME) and homogenized with stainless steel beads using a TissueLyzer II instrument (QIAGEN). Total RNA was purified using an RNeasy Mini Kit (QIAGEN) according to the manufacturer’s instructions. Real-time qRT-PCR was performed as described previously^23^. Briefly, total RNA was isolated from body fluid samples (40 uL serum, 140 uL urine, and 140 uL saliva) using a QIAmp Viral RNA Mini Kit (QIAGEN) and eluted in 60 uL. For fluid samples, 5 uL of extracted RNA was used and for tissue samples, 1 μg of total RNA was used in a duplicate 20 µL reaction with TaqMan Fast Virus 1-Step Master Mix (Thermo-Fisher Scientific). RT was performed at 50 °C for 15 min followed by inactivation/denaturation at 94 °C for 2 min then thermocycling up to 45 cycles of denaturation at 95 °C for 15 s and polymerization at 59 °C for 30 s was performed on a 7500 Fast Real-time PCR System (Applied Biosystems) The sequence of ZIKV-specific primers and probe were previously described^46^: forward, 5′-CCGCTGCCCAACACAAG-3′, reverse 5′-CCACTAACGTTCTTTTGCAGACAT-3′, probe 5′-/56-FAM/AGCCTACCT/ZEN/TGACAAGCAGTCAGACACTCAA/3IABkFQ/-3′ (Integrated DNA Technologies). The PCR condition was optimized using 1 ug total RNA per reaction. The following controls were included in each RT-PCR run: five points of ZIKV RNA standard curve, two quality control samples with Ct values of 26 and 32, and no template control, all in duplicate. ZIKV RNA levels were interpolated against standard curves prepared by diluting RNA from uninfected tissue spiked with known copy numbers of ZIKV genomic RNA (NR-50244, BEI Resources) in 1 ug total RNA from tissues from naïve animals. Under the optimized condition, we defined the limit of detection (LOD) as Ct value equal to 37 with 70% positivity over 20 independent PCR runs. The Ct value 37 corresponds to 1.3 copies of viral RNA per 1 ug RNA. The assay limit of quantitation (LOQ) was defined as the Ct value equal to 35 with 100% positivity, which is equivalent to 5.8 copies per 1 ug RNA. Hence, Ct values < 35 were used to calculate the copy number per gram of tissue or mL body fluid samples. The Ct values > 35 were considered as below the limit of quantification (BLQ) because infrequent low-level viral RNA was impossible to quantitate reliably.

### Deep sequencing analysis of ZIKV cultures

Nucleic acids were extracted from stock vials of the 2^nd^ passage, the original virus stock from Dr. Lark Coffey, and the 4^th^ passage of ZIKV-BR SPH2015 (propagated at Trudeau institute), using the Zymo Direct-zol RNA (Zymo Research) miniprep kit using manufacturer guidelines, followed by treatment with Turbo DNase (Thermo-Fisher, Carlsbad, CA). cDNA was obtained by reverse transcription using a 1:5 mix of random hexamer and short spiked primers targeting ZIKV genomes^47^. Next-generation sequencing (NGS) libraries were constructed using the Nextera XT kit (Illumina, San Diego, CA), followed by single-end, 150 base pair (bp) sequencing on an Illumina MiSeq or HiSeq. 2500 instrument. Raw reads were preprocessed by adapter trimming, and removal of low-quality and low-complexity sequences as previously described^48^. Data were scanned for ZIKV reads using an NCBI BLAST database constructed from all known ZIKV sequences as of October 2017, using an *E*-value significance threshold of 1 × 10^−5^. Consensus ZIKV genomes were assembled using Geneious (v10.2.2) by mapping preprocessed ZIKV reads to reference genome KU321639^49^.

### Detection of infectious virus particles in the placentas of marmosets using U937 cells

Frozen placentas obtained at 14 dpi were thawed in ice. Placentas were cut into multiple pieces. Randomly selected pieces from 3–4 different sites were weighed and then placed in sterile tubes containing in RPMI-1640 medium supplemented with 2% FBS and homogenized using TissueLyzer for 1 min. Cell debris was cleared by centrifugation at 20,000 × g for 5 min. Supernatant was removed and serially diluted in the medium. As a positive control, the mouse monoclonal antibody clone ZK78 at the concentration of 200 ng/mL, which was previously optimized to cause 10–15% ADE, was used to co-culture with ZK-PR at M.O.I of 1.26. Homogenate of the placenta from marmoset which was infected with DENV prior to ZIKV infection during pregnancy (confirmed vRNA loads at 6.5 × 10^9^ copies per gram tissue) was included in the assay as a positive control. Medium containing virus only was used as a negative control. U937 cells were adjusted at the concentration of 5 × 10^6^ cells/mL. Fifty uL of U937 cells were added to each well of the U-bottomed 96-well plate. Dilutions of placental homogenate and controls were incubated at 37 °C for 2 h then 100uL of each condition were added in to U937 cells containing plates in duplicate and plates were incubated overnight at 37 °C supplemented with 5% CO_2_. The plates were washed with 1x PBS containing 0.05% Tween 20 (PBST buffer) four times. Then, the Env protein of ZIKV on U937 cells was stained using pan-flavivirus monoclonal antibody (clone 4G2)-conjugated with PE. After washing cells, the cells were fixed in PBS containing 1% paraformaldehyde, acquired using FACS Canto II (BD Biosciences, San Jose, CA), and analyzed by using Flow-Jo software (version 10). The presence of the Env protein of ZIKV in U937 cells is considered as infection of naïve U937 cells via Fc-receptor-mediated infection. Fold-increase was calculated as percent positive to 4G2 of U937 cells containing placental homogenate per well divided by percent positive to 4G2 of U937 cells containing medium only.

### Statistical analyses

Statistical analyses were performed using GraphPad Prism Software v.8 (San Diego, CA). Contingency data of the percent fetal demise and protection were analyzed using Fisher’s exact test. Viral RNA loads and neutralizing antibody titers between groups were analyzed using the ANOVA test. Spearman’s *r*-test was used to analyze the correlation between neutralizing antibody titers and the percent of fetal protection per dam as well as the correlation between neutralizing antibody titers and ZIKV RNA loads in the maternal spleen.

### Reporting summary

Further information on research design is available in the Nature Research Reporting Summary linked to this article.

## Supplementary information

Reporting Summary

Supplementary Information

## Data Availability

All The data generated and analyzed during the current study are available from the corresponding authors upon reasonable request.
