# Author Correction: Efficacy of an inactivated Zika vaccine against virus infection during pregnancy in mice and marmosets

**DOI:** 10.1038/s41541-022-00520-x

**Published:** 2022-08-20

**Authors:** In-Jeong Kim, Paula A. Lanthier, Madeline J. Clark, Rafael A. De La Barrera, Michael P. Tighe, Frank M. Szaba, Kelsey L. Travis, Timothy C. Low-Beer, Tres S. Cookenham, Kathleen G. Lanzer, Derek T. Bernacki, Lawrence L. Johnson, Amanda A. Schneck, Corinna N. Ross, Suzette D. Tardif, Donna Layne-Colon, Stephanie D. Mdaki, Edward J. Dick, Colin Chuba, Olga Gonzalez, Kathleen M. Brasky, John Dutton, Julienne N. Rutherford, Lark L. Coffey, Anil Singapuri, Claudia Sanchez San Martin, Charles Y. Chiu, Stephen J. Thomas, Kayvon Modjarrad, Jean L. Patterson, Marcia A. Blackman

**Affiliations:** 1 Trudeau Institute, Inc., Saranac Lake, NY 12983 USA; 2 Pilot Bioproduction Facility, Center for Enabling Capabilities, Walter Reed Army Institute of Research, Silver Spring, MD 20910 USA; 3 Southwest National Primate Center, Texas Biomedical Research Institute, San Antonio, TX 78227 USA; 4 Department of Human Development Nursing Science, College of Nursing, University of Illinois Chicago, Chicago, IL 60612 USA; 5 Department of Pathology, Microbiology and Immunology, School of Veterinary Medicine, University of California, Davis, CA 95616 USA; 6 Department of Laboratory Medicine, School of Medicine, University of California at San Francisco, San Francisco, CA 94158 USA; 7 Division of Infectious Diseases, Institute for Global Health and Translational Sciences, State University of New York, Upstate Medical University, Syracuse, NY 13210 USA; 8 Emerging Infectious Diseases Branch, Walter Reed Army Institute of Research, Silver Spring, MD 20910 USA; 9 Present Address: Division of Infectious Diseases and Vaccinology, School of Public Health, University of California at Berkeley, Berkeley, CA 94720 USA

**Keywords:** Viral infection, Viral infection

Correction to: *npj Vaccines* 10.1038/s41541-021-00426-0, published online 27 January 2022

In the original version of this Article, in Fig. 1e, the number above the mock group bar shows 1/3. The correct number should have been 2/3. This has now been corrected in both the HTML and PDF versions of this Article.

