# Supplementary material for: Efficacy of an inactivated Zika vaccine against virus infection during pregnancy in mice and marmosets
Source: NPJ Vaccines. 2022 Jan 27;7:9. doi: 10.1038/s41541-021-00426-0 (PMC8795414; doi:10.1038/s41541-021-00426-0)
Supplement: Supplementary file 1 — Reporting Summary [file 41541_2021_426_MOESM1_ESM.pdf]

nature portfolio

Corresponding author(s): Marcia A. Blackman, Jean L. Patterson, In-Jeong Kim

Last updated by author(s): November 15, 2021

Reporting Summary

Nature Portfolio wishes to improve the reproducibility of the work that we publish. This form provides structure for consistency and transparency in reporting. For further information on Nature Portfolio policies, see our Editorial Policies and the Editorial Policy Checklist.

Statistics

For all statistical analyses, confirm that the following items are present in the figure legend, table legend, main text, or Methods section.

n/a	Confirmed	
☐	☒	The exact sample size (n) for each experimental group/condition, given as a discrete number and unit of measurement
☐	☒	A statement on whether measurements were taken from distinct samples or whether the same sample was measured repeatedly
☐	☒	The statistical test(s) used AND whether they are one- or two-sided<br>*Only common tests should be described solely by name; describe more complex techniques in the Methods section.*
☐	☒	A description of all covariates tested
☐	☒	A description of any assumptions or corrections, such as tests of normality and adjustment for multiple comparisons
☐	☒	A full description of the statistical parameters including central tendency (e.g. means) or other basic estimates (e.g. regression coefficient) AND variation (e.g. standard deviation) or associated estimates of uncertainty (e.g. confidence intervals)
☐	☒	For null hypothesis testing, the test statistic (e.g. F, t, r) with confidence intervals, effect sizes, degrees of freedom and P value noted<br>*Give P values as exact values whenever suitable.*
☒	☐	For Bayesian analysis, information on the choice of priors and Markov chain Monte Carlo settings
☒	☐	For hierarchical and complex designs, identification of the appropriate level for tests and full reporting of outcomes
☐	☒	Estimates of effect sizes (e.g. Cohen's d, Pearson's r), indicating how they were calculated

Our web collection on statistics for biologists contains articles on many of the points above.

Software and code

Policy information about availability of computer code

Data collection	N.A
Data analysis	GraphPad Prism Software v.8

For manuscripts utilizing custom algorithms or software that are central to the research but not yet described in published literature, software must be made available to editors and reviewers. We strongly encourage code deposition in a community repository (e.g. GitHub). See the Nature Portfolio guidelines for submitting code & software for further information.

Data

Policy information about availability of data

All manuscripts must include a data availability statement. This statement should provide the following information, where applicable:

- Accession codes, unique identifiers, or web links for publicly available datasets
- A description of any restrictions on data availability
- For clinical datasets or third party data, please ensure that the statement adheres to our policy

All data presented in this publication are available upon reasonable requests to corresponding authors.

Field-specific reporting

Please select the one below that is the best fit for your research. If you are not sure, read the appropriate sections before making your selection.

☒ Life sciences ☐ Behavioural & social sciences ☐ Ecological, evolutionary & environmental sciences

For a reference copy of the document with all sections, see nature.com/documents/nr-reporting-summary-flat.pdf

Life sciences study design

All studies must disclose on these points even when the disclosure is negative.

Sample size	Based on the results from the previous mouse study (Szaba et al., 2018), we intravenous inoculation of pregnant C57BL/6 mice at E9.5 with 5 x10^5 PFU of Zika virus caused approximately 70% fetal demise. Assuming ZPIV vaccination confers 100% protection, then to achieve 80% power at a significant level of p=0.05, calculation indicates we will need 6 mice per treatment. Not all plug detected female mice become pregnant. Previously, we have achieved approximately 80 % of pregnancy in the timed pregnancy studies. In order to achieve 6 pregnant females per treatment, 8 females will be co-housed with males in a ratio of 2:1.<br><br>Because of the availability of published data (Seferovic et. al., 2018) from ZIKV infected pregnant marmosets, we examined 1 unvaccinated marmoset and 4 vaccinated marmosets were examined. Marmosets bear multiple births with each pregnancy increasing the probability to detect statistical differences between different treatments. The litter size in captive marmosets varies from 2-4 per pregnancy with an average of 2.5 fetuses per litter. Previous data suggested that in order to achieve 80% power to detect a difference in viral loads at a significance level of p=0.05, we would need 6 fetuses requiring a minimum of 3 pregnant marmosets per group.
Data exclusions	No data were excluded for data analysis.
Replication	Two independent experiments were performed in C57BL/6 mice with the two-dose vaccination regimen (n=6-8 per group). The marmoset study was not repeated.
Randomization	Age comparable mice (6 weeks old) and marmosets (2-2.5 years old) were randomly assigned for the experimental groups.
Blinding	N.A.

Reporting for specific materials, systems and methods

We require information from authors about some types of materials, experimental systems and methods used in many studies. Here, indicate whether each material, system or method listed is relevant to your study. If you are not sure if a list item applies to your research, read the appropriate section before selecting a response.

Materials & experimental systems

n/a	Involved in the study	
☒	☐	Antibodies
☐	☒	Eukaryotic cell lines
☒	☐	Palaeontology and archaeology
☐	☒	Animals and other organisms
☒	☐	Human research participants
☒	☐	Clinical data
☒	☐	Dual use research of concern

Methods

n/a	Involved in the study	
☒	☐	ChIP-seq
☐	☒	Flow cytometry
☒	☐	MRI-based neuroimaging

Eukaryotic cell lines

Policy information about cell lines

Cell line source(s)	Vero cells were purchased from American Tissue and Cell Culture
Authentication	Vero (ATCC CCL-81)
Mycoplasma contamination	Vero cell culture supernatant was tested for Mycoplasma pulmonis and other Mycoplasma species, prior to virus-replication in vitro and the result confirmed to be free of the Mycoplasma.(IMPACT test Case # 24231-2016 by IDEXX BioResearch)
Commonly misidentified lines (See ICLAC register)	N.A.

Animals and other organisms

Policy information about studies involving animals; ARRIVE guidelines recommended for reporting animal research

Laboratory animals	Six weeks old female C57BL/6J mice from JAX were used. Common marmosets (Callithrix jacchus) were bred and maintained in the Southwestern National Primate Research Center (SNPRC)
Wild animals	N.A.
Field-collected samples	N.A.
Ethics oversight	The mouse studies were conducted following the approved IACUC protocol 19-003 by the Trudeau Institute IACUC committee and the marmoset studies were conducted following the approved IACUC protocol 1528CJ by the SNPRC and Texas Biomedical Research Institute. All animal studies were approved by by the Animal Care and Use Review Office (ACURO) at the Department of Defense.

Note that full information on the approval of the study protocol must also be provided in the manuscript.

Flow Cytometry

Plots

Confirm that:

- [ ] The axis labels state the marker and fluorochrome used (e.g. CD4-FITC).
- [ ] The axis scales are clearly visible. Include numbers along axes only for bottom left plot of group (a 'group' is an analysis of identical markers).
- [ ] All plots are contour plots with outliers or pseudocolor plots.
- [x] A numerical value for number of cells or percentage (with statistics) is provided.

Methodology

Sample preparation	Dilutions of placental homogenate and controls with virus were incubated at 37°C for 2 hrs then 100uL of each condition were added in to U937 cells containing plates in duplicate and plates were incubated overnight at 37°C supplemented with 5% CO2. The plates were washed with 1XPBS containing 0.05% Tween 20 (PBST buffer) 4 times. Then, the Env protein of ZIKV on U937 cells was stained using pan –flavivirus monoclonal antibody (clone 4G2)-conjugated with PE. After washing cells, the cells were fixed in PBS containing 1% paraformaldehyde, acquired on a flow cytometric instrument.
Instrument	FACS Canto II
Software	Flow cytometry data were acquired on FACS Canto II using the BD FACSDIVA software (BD biosciences, San Jose, CA) and analyzed by using Flow-Jo software v. 10 (TreeStar Inc., CA).
Cell population abundance	Human monocyte, U937 cells are sole population (100%) of FACS analysis
Gating strategy	1. Single cells were gated based on FSC-A against FSC-H<br>2. Dead cell debris were gated out and the monocyte population was gated on based of FSC-A and SSC-A<br>3. 4G2-PE-positive signal was gated higher than the signal from the cells stained with isotype control IgG

- [ ] Tick this box to confirm that a figure exemplifying the gating strategy is provided in the Supplementary Information.
