# Supplementary material for: Efficacy of an inactivated Zika vaccine against virus infection during pregnancy in mice and marmosets
Source: NPJ Vaccines. 2022 Jan 27;7:9. doi: 10.1038/s41541-021-00426-0 (PMC8795414; doi:10.1038/s41541-021-00426-0)
Supplement: Supplementary file 2 — Supplementary Information [file 41541_2021_426_MOESM2_ESM.pdf]

Supplementary Information

Efficacy of an inactivated Zika vaccine against virus infection during pregnancy in mice and marmosets

In-Jeong Kim[1*], Paula A. Lanthier[1], Madeline J. Clark[1], Rafael A. De La Barrera[2], Michael P. Tighe[1], Frank M. Szaba[1], Kelsey L. Travis[1], Timothy C. Low-Beer[1], Tres S. Cookenham[1], Kathleen G. Lanzer[1], Derek T. Bernacki[1], Lawrence L. Johnson[1], Amanda A. Schneck[1], Stephanie D. Mdaki[3], Corinna N. Ross[3], Suzette D. Tardif [3], Donna Layne-Colon[3], Edward J. Dick Jr[3], Colin Chuba[3], Olga Gonzalez[3], Kathleen M. Brasky[3], John Dutton[3], Julienne N. Rutherford[4], Lark L. Coffey[5], Anil Singapuri[5,] Claudia Sanchez San Martin[6,9], Charles Y. Chiu[6], Stephen J. Thomas[7], Kayvon Modjarrad[8], Jean L. Patterson[3*], Marcia A. Blackman[1*].

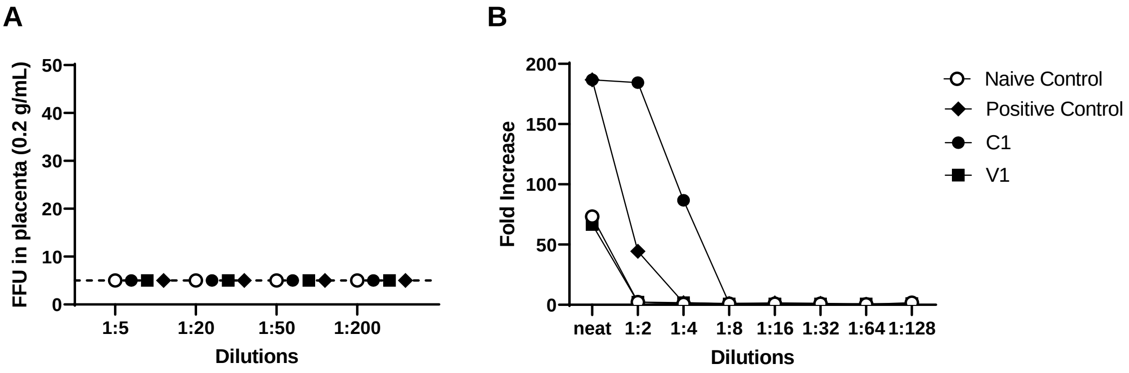

Supplementary Figure 1. Infectious virus particles were not detectable in vaccinated marmosets

The placentas from marmosets harvested at 14 dpi were homogenized, diluted, and co-cultured with either Vero cell monolayers for 48 hrs or U937 cells overnight at 37 °C. A. Focus-forming assay. B. Antibody-mediated infection of U937 cells. Note that infectious virus particles in the unvaccinated ZIKV challenged marmoset C1 were undetectable by focus-forming assay but were detectable by antibody-mediated infection. The vaccinated marmoset V1 containing detectable viral RNA (Table 2) was comparable to the background level of the naïve marmoset, consistent with no infectious virus particles and indicating that the PCR product may be derived from fragments of viral genome from destroyed viruses. The gestationally comparable placenta from naïve marmoset was used as a negative control and a placenta that was previously tested positive for infectious virus particles was used as a positive control. Results represent two independent assays performed on different days.

Supplementary Table 1. Comparison of relative efficacy between single-dose and two-dose vaccination in mice

Group	Number of fetuses			% Affected fetus	% Protected fetus	Relative efficacy[1]
	Affected	Normal	Total			
Alum (1x)	24	12	36	66.7%	33%	
ZPIV(1x)	11	69	80	13.8%	86%	79.4%
Alum (2x)	54	22	76	71.1%	29%	
ZPIV(2x)	7	46	53	13.2%	87%	81.4%

[1] Relative efficacy was calculated as the percent reduction of the rate of fetal abnormality of the vaccinated group relative to the rate of fetal abnormality of the unvaccinated group.

The relative efficacy between one dose and two-dose vaccinations was not significantly different ($P > 0.05$).

Supplementary Table 2. Comparison of relative protective efficacy after challenge with ZIKV strains in mice

Group	Number of fetuses			% Affected fetus	% Protected fetus	Relative efficacy
	Affected	Normal	Total			
Alum-PR	49	11	60	81.7%	18%	
ZPIV-PR	8	57	65	12.3%	88%	84.9%
Alum-BR	87	28	115	75.7%	24%	
ZPIV-BR	10	118	128	7.8%	92%	89.7%

Supplementary Table 3. Virus neutralizing antibody (log MN_{50}) titers[1] in C57BL/6 mice after ZPIV vaccination

	Alum (AlOH, 2 doses) and challenged with ZIKV-PR week post-Vx[2]					Alum (AlOH, 2 doses) challenged with ZIKV-BR week post-Vx[2]				
Timepoint	0[3]	2	6	7[4]	8[5]	0[3]	2	6	7[4]	8[5]
	0.70	0.70	0.70	0.70	1.81	0.70	0.70	0.70	0.70	2.91
	-	0.70	0.70	0.70	0.70	-	0.70	0.70	0.70	3.06
	-	0.70	0.70	0.70	2.21	-	0.70	0.70	0.70	2.90
	-	0.70	0.70	0.70	2.57	-	0.70	0.70	0.70	2.39
	-	0.70	0.70	0.70	2.49	-	0.70	0.70	0.70	2.72
	-	0.70	0.70	0.70	2.55	-	0.70	0.70	0.70	2.37
	-	0.70	0.70	0.70	2.75	-	-	-	-	-
	-	0.70	0.70	0.70	1.78	-	-	-	-	-
GMT[7]	0.70	0.70	0.70	0.70	2.11	0.70	0.70	0.70	0.70	2.73
95% C.I.[8]	N.A.	0.70	0.70	0.70	(1.4 - 2.5)	N.A.	0.70	0.70	0.70	(2.4 - 3.0)
	ZPIV (2 doses) challenged with ZIKV-PR week post-Vx[2]					ZPIV (2 doses) challenged with ZIKV-BR week post-Vx[2]				
Timepoint	0[3]	2	6	7[4]	8[5]	0[3]	2	6	7[4]	8[5]
	0.70	2.38	3.67	3.50	3.15	0.70	1.92	3.56	3.17	3.77
	-	0.70	3.12	2.97	3.07	-	2.18	3.86	3.55	3.48
	-	1.49	3.08	2.23	3.10	-	2.39	3.86	3.86	3.73
	-	1.18	2.86	2.64	3.08	-	1.26	3.69	3.23	4.40
	-	1.28	3.86	3.81	3.76	-	1.23	3.86	3.86[6]	4.23
	-	1.81	3.22	3.00	3.01	-	1.38	3.86	3.86[6]	4.17
	-	1.67	3.86	3.86[f]	4.19	-	1.26	3.86	3.44	3.36
	-	1.04	2.75	3.10	3.95	-	1.34	3.66	3.86[6]	3.89
	-	1.23	3.86	3.86[f]	4.55	-	1.59	3.49	2.75	4.17
	-	1.67	3.27	3.86[f]	3.75	-	-	-	-	-
GMT[7]		1.45	3.36	3.28	3.52		1.61	3.75	3.51	3.90
95% C.I.[8]	N.A.	(1.0 - 1.7)	(3.0 - 3.6)	(2.8 - 3.7)	(3.2 - 3.9)	N.A.	(1.2 - 1.9)	(3.6 -3.9)	(3.2 -3.8)	(3.6 -4.2)

[1] MN50 titers are presented as the log-transformed reciprocal serum dilution achieving 50% virus neutralization

[2] Mice were vaccinated at week 0 and week 4

[3] Prior to vaccination, serum samples were prepared and an equal volume from individual mice was pooled and tested.

[4] MN_{50} titer determined at E8.5, one day prior to ZIKV challenge.

[5] MN_{50} titer determined at E17.5, 8 dpi.

[6] The last serum dilution tested achieved greater than 50% neutralization.

[7] GMT, Geometric mean

[8] C.I., Confidence interval

Supplementary Table 4. Frequency of mutations of the ZIKV-BR SPH2015 stocks

ZIKV-BR SPH2015 origin/passage	Location in	UC Davis/P2	Trudeau/P4
Infected animals in the study	ZIKV protein	Marmoset	C57BL/6
Mutation position:			
859 (T>C, UUC to CUC) (F252L)	M	0.3 %	19.5%
3533 (T>A, AUG to AAG) (M1143K)	NS1	2 %	21.4%
4317 (G>A, AUG to AUA) (M1404I)	NS2B	1 %	10.6%
Random control at 6117	NS3	0.1- 0.5 %	0.0%

Sequence analysis of the ZIKV-BR SPH2015 inoculum

F252L is located in the structural membrane protein. This mutation was also present in the microcephaly case that had M1143K, but F252L has not been present in macaque, nor associated to microcephaly or another condition [1].

F252L mutation was detected in one of two marmosets that miscarried fetuses [2]. F252L is considered as a marmoset-specific mutation. M1401I is located in the NS2B protein of ZIKV. M1401I was detected in a human case with microcephaly but is not in most genomes from outbreaks since 2015, evolved *de novo* in rhesus macaques, and confers fetal infection in a pregnant CD-1 mouse model (GenBank PRJNA556052) [3]. Both the F252L and M1401I mutations were detected in another human case (GenBank number KU870645). Throughout this study, we have not detected any noticeable differences in fetal abnormalities from the Puerto Rican strain of ZIKV, PRVABC59 (Gene Bank Accession Number KU501215.1). Because it is outside of the study scope, whether the high frequency of F252L and M1401I mutations in the P4 virus stocks used for the mouse studies alter ZIKV pathogenesis in immunocompetent mice was not examined. The effect of these mutations on ZIKV pathogenesis in mice may require side-by-side comparison with wild type ZK-BR in further investigations.

Supplementary References

1 Driggers, R. W. *et al.* Zika Virus Infection with Prolonged Maternal Viremia and Fetal Brain Abnormalities. *N Engl J Med* **374**, 2142-2151, doi:10.1056/NEJMoa1601824 (2016).

2 Seferovic, M. *et al.* Experimental Zika Virus Infection in the Pregnant Common Marmoset Induces Spontaneous Fetal Loss and Neurodevelopmental Abnormalities. *Sci Rep* **8**, 6851, doi:10.1038/s41598-018-25205-1 (2018).

3 Lemos, D. *et al.* Two sides of a coin: a Zika virus mutation selected in pregnant rhesus macaques promotes fetal infection in mice but at a cost of reduced fitness in nonpregnant macaques and diminished transmissibility by vectors. *J Virol*, doi:10.1128/JVI.01605-20 (2020).
